# Evaluation of the clinical effectiveness of modified sacrospinous ligament fixation *via* the anterior vaginal wall path for pelvic organ prolapse: A feasibility report based on 50 patients

**DOI:** 10.3389/fsurg.2022.1010027

**Published:** 2022-11-02

**Authors:** Yuanyuan Lyu, Huafeng Ding, Ying Zhang, Suhua Shi, Jin Ding, Chengcheng Zhu, Xiaoming Guan, Guantai Ni, Yonghong Luo

**Affiliations:** ^1^ Department of Obstetrics and Gynecology, The First Affiliated Hospital of Wannan Medical College, Wuhu, China; ^2^ Department of Obstetrics and Gynecology, Baylor College of Medicine Minimally Invasive Gynecology Surgery, Houston, TX, United States

**Keywords:** modified sacrospinous ligament fixation, T4, pelvic organ prolapse, surgery, mesh

## Abstract

**Objective:**

To describe the surgical techniques and short-term outcomes for 50 cases of modified sacrospinous ligament fixation *via* the anterior vaginal wall path for pelvic organ prolapse

**Methods:**

100 patients with pelvic organ prolapse (stage III or stage IV based on POP-Q staging) from January 2018 to January 2020 were retrospectively analyzed. Among them, 50 patients received modified sacrospinous ligament fixation *via* the anterior vaginal wall path for pelvic organ prolapse (mSSLF group), while the other 50 patients received pelvic reconstruction using T4 mesh (T4 group). Operative time, blood loss, postoperative POP-Q score, length of the hospital stay, complications, and postoperative pain were compared between the two groups.

**Results:**

The duration of the operation in mSSLF group was (50 ± 15.2 min), which was shorter than that of the T4 group (60 ± 14.8 min) (*p *= 0.02). No intraoperative complications were reported from the mSSLF group, whereas one vascular injury occurred in the T4 group. In both groups, postoperative pain and painful intercourse was significantly lower in the mSSLF group than in the SSLF group (*p* < 0.001). The exposed mesh rate was lower than T4 group.

**Conclusions:**

The rates of intraoperative complications, postoperative pain and mesh erosion were significantly lower than those of the T4 group, but there was no significant difference in the efficacy and safety of the treatment of pelvic organ prolapse. So mSSLF may be a feasible technique to manage severe prolapse, with promising short-term efficacy and safety.

## Introduction

Pelvic organ prolapse (POP) is a group of common gynecological diseases caused by weakened pelvic floor support tissues, resulting in the prolapse and displacement of pelvic organs (1, 2). For moderate and severe POP, surgery is the main method of treatment (3). Traditional repair surgery results in a high recurrence rate, many complications, and a high level of damage. Especially the recurrence rate of anterior vaginal wall prolapse after sacrospinous ligament fixation (SSLF) can be as high as 20% and result in postoperative hip pain related complications (4). The open or laparoscopic approach requires opening the retropubic space or the retroperitoneum on both sides of the anterior sacrum to expose the sacrospinous ligament, which can easily damage the ureter and vascular plexus, resulting in ureteral injury or bleeding (5, 6).

During recent years, progress in female pelvic floor anatomy related research has led to the “holistic theory” and “three levels of the vagina” (7, 8), as well as improvement in surgical instruments and repair materials used, while various novel procedures have emerged with the aim of reconstructing the pelvic floor structure and restoring pelvic floor function (9). The T4 mesh pelvic floor reconstruction method that is simple, shortens operation time, has a small scope, is less invasive, reduces intraoperative bleeding, allows patients to recover quickly following surgery, and can be considered as a novel minimally invasive surgery. However, since this pelvic floor repair method requires extracorporeal puncture to introduce the mesh band, challenges, such as postoperative pain in the puncture area and tissue damage, are frequent. In addition, the most common problem encountered is mesh erosion (10).

Inspired by the Prosima procedure, T4, and classic sacrospinous ligament fixation, we developed an innovative method of sacrospinous ligament fixation that only requires the use of a small amount of mesh. This procedure not only increases the contact area between the vaginal apex and the sacrospinous ligament, but also reduces the intraoperative separation area and allows the mesh to be placed in a tension-free state, thus solving the problems of high postoperative vaginal wall tension, perineal pain and puncture pain in the traditional procedure. In addition, it uses less mesh, which significantly reduces the complications of postoperative mesh exposure. The procedure is simple, minimally invasive, and reproducible, with the goal of strengthening the pelvic floor anatomy and thus restoring pelvic floor function.

## Materials and methods

Preoperative examination: patients underwent routine general and gynecological examinations before surgery, including routine blood and urine tests, coagulation series, infectious indexes, electrocardiogram, and chest x-ray to assess the function of important organs and whether they will be able to tolerate surgery. If necessary, urological ultrasound and rectal examinations were also performed to rule out the presence of anal and rectal lesions. Before pelvic examination, the patient was requested to empty their bladder, assume the lithotomy position, and the most severe degree of uterine prolapse was determined using forceful downward breath-holding or abdominal pressure, while the Pelvic Organ Prolapse Quantitation (POP-Q) was applied to assess the degree of prolapse. In addition, (1) For some patients with pelvic tissue prolapsing outside the vaginal orifice, where long-term friction had led to redness, erosion, ulceration and infection, 1:5,000 potassium permanganate sitz was added to the bath 1.2 times a day for 15–30 min, and wait for infection control and ulcer healing before proceeding to surgical treatment; (2) Intestinal preparation: administer an oral laxative, such as magnesium sulfate, after fasting for 8 h and water for 4 h before surgery; (3) Preoperative education: Communicate fully with the patient and his family about the necessity of surgery, risks involved, as well as intraoperative and postoperative complications. And we re-evaluated each patient's prolapse after anesthesia.

For this case-control study, data were obtained from patient electronic medical ﬁle records. We reviewed ﬁles of 100 women who underwent surgery by either the mSSLF or the T4 approach between 2018 and 2020. These patients were not included in previous published papers. The inclusion criteria were as follows: (1) age 25–79 years; (2) severe prolapse (≥ stage 3); (3) desire for preservation of coital function; (4) first surgical treatment for POP; and The exclusion criteria were as follows: (1) inability to tolerate surgery; (2) coagulation dysfunction; (3) severe vaginal ulcers; (4) inability to tolerate the Trendelenburg position;(5) suspicion of gynaecological malignancy.

### Surgical method

In the study group, 50 patients underwent modified pelvic floor reconstruction using a X-mesh, while 50 other patients underwent T4-mesh pelvic floor reconstruction. Differences in the characteristics of the patients, such as age, age at menopause, and number of pregnancies and deliveries, between the two groups were not statistically significant (*p* > 0.05) and were comparable, as shown in Table 1.

**Table 1:** Characteristics of the study patients (*n* = 100).^a^

Characteristics	mSSLF group (*n* = 50)	T4 group (*n* = 50)	*p-*value
Age, y	65 ± 5.2	66 ± 4.8	0.38
Stress urinary incontinence	3	4	0.69
Hysterectomy	34	29	0.13
Forceps/vacuum delivery, time	3.10 ± 2.10	3.30 ± 2.10	0.32
Menopausal, y	46	48	0.743
Preoperative POP-Q			
Aa	2.6 ± 0.3	2.5 ± 0.3	0.71
Ba	4.15 ± 0.6	4.4 ± 0.62	0.31
Ap	2.2 ± 0.3	2.3 ± 0.3	0.28
Bp	3.4 ± 0.9	3.5 ± 1.0	0.52
C	6.0 ± 1.2	6.3 ± 1.3	0.25
TVL	8.3 ± 0.2	8.5 ± 0.2	0.34
POP stage before operation			
III	38	36	0.65
IV	12	14	0.65

^a^ Values are given as mean ± SD, number, number (percentage), or median (range), unless indicated otherwise.

### T4 mesh pelvic floor reconstruction

After anesthesia, the bladder was catheterized and emptied, and the vagina was incised below the external urethra. The mucosa of the anterior vaginal wall was incised 1 cm below the urethral orifice. A skin incision was made 2 cm outside and 2 cm above the genital folds on both sides of the vulva, and a special puncture needle was used to pass along the butterfly guide rod. The pelvic fascia was penetrated from the level of the closed fossa and the sciatic spine, respectively. The puncture needle was removed. Adjust the position of the mesh band, suture the pericervical. The paracervical primary ligament tissue is sutured around the cervix, and the posterior midpoint of the mesh is fixed to ensure that the mesh is tension-free.

### mSSLF

**Figure 1:**
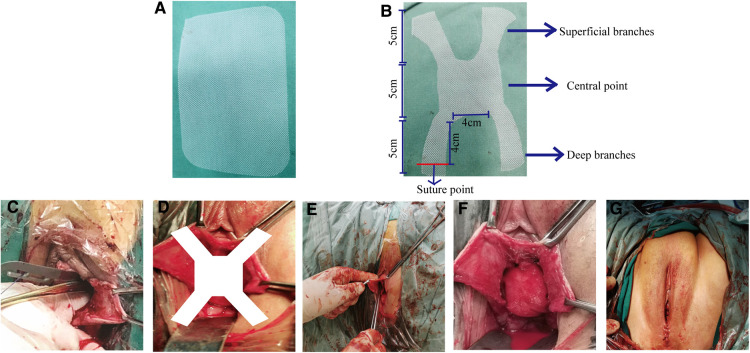
(**A**) The 10*15 cm original mesh. (**B**) Schematic diagram of the X-shaped mesh after construction. (**C**) Separation of the sacrospinous ligament. (**D**) Schematic diagram of X-mesh placemented in the body. (**E**) Fixed the mesh in the body. (**F**) Diagram of the complete placement of the mesh into the bod. (**G**) Postoperative of mSSLF.

Trim the 15 cm×10 cm mesh (Figure 1A) into an “X” shape (Figure 1B). Longitudinal incision of the anterior vaginal wall *via* the apical midline of the vagina using a combination of blunt and sharp separation techniques from the vaginal mucosa to the primary bilateral sciatic spine/sacral spine ligaments. The deep branches ohe X-mesh were fixed separately to the ipsilateral sacrospinous ligament using PDS sutures. The central point suture was made anterior to the cervix (preservation of the uterus) or anterior to the main-sacral ligament complex (removal of the uterus). The superficial branch was placed in the interstitial space between the subvaginal mucosa on both sides of the bladder by subducting it to the obturator membrane of the descending pubic symphysis. The vaginal incision was kept flat and tension-free, and was sutured after the operation, while iodine gauze was placed inside the vagina(See in Figure 1).

### Intra-operative and post-operative evaluation

The parameters related to the surgery, such as the operation time, intraoperative bleeding and the presence of peripheral organ damage, were recorded. Indicators of postoperative recovery, such as the duration of ureteral retention, postoperative hospitalization days, postoperative morbidity, urinary retention, and other complications, and their management were also recorded. Transvaginal hysterectomy for patients with uterine lesions (fibroids, polyps, etc.) and patients with hysterectomy hospitals. Intraoperative transvaginal closed-hole tension-free midurethral suspension(TVT-O) was performed in patients with preoperative stress urinary incontinence.

### Follow-up and assessment

POP-Q staging assessment was performed. The clinical efficacy follow-up was conducted as follows: (1) Self-perceived symptoms: The patients were evaluated using subjective sensation and were asked whether they had any postoperative symptoms, such as pain, increased vaginal discharge, urinary discomfort, including urinary incontinence, abnormal defecation, lower limb pain, and vaginal detrusor. (2) Pelvic examination: the healing of the vaginal incision was observed, with the presence of infection and mesh erosion exposure evaluate based on POP-Q staging. A degree of I was designated if objective anatomical cure was observed, while a degree of II was used to record recurrence.

### Statistical analysis

Data collection and statistical analyses were performed using IBM SPSS Statistics 22.0 software (IBM Corp. Armonk, New York, USA). All variables are presented as the mean and standard deviation (SD) or n and percentage (%). Continuous variables were compared by Student's *t*-test. A *p* value <0.05 was considered statistically signifcant.

## Results

### Comparison of intraoperative conditions between the two groups

The intraoperative conditions were compared between the observation group and the control group. The operative time in the mSSLF group was 50 ± 15.2 min, which is shorter than that of the T4-mesh group at 60 ± 14.8 min (*p *= 0.02). In addition, the total amount of intraoperative bleeding in the mSSLF group was 70 ± 10.2 ml, which is also significantly less than that of the T4-mesh group at 90 ± 14.8 ml (*p* = 0.03) due to less intraoperative separation gap. The operations of all patients were smooth and there were no intraoperative side injuries recorded to the bladder or rectum, except for one case of bleeding (600 ml) due to a vascular injury that occurred during the separation of the vaginal bladder space in the T4 mesh group, which improved after compression therapy was provided. There were also no cases of serious complications, such as delayed bleeding, infection, or abscess after surgery until the patient was discharged. There were also no statistically significant differences observed between the two groups based on the time to remove the urinary catheter after surgery, and postoperative hospital stay (*p* > 0.05). The mean duration of continuous catheterization in the T4 group was 4.4 ± 1.1 days, while the mean duration of retained urinary catheter in the mSSLF group was 3.98 ± 0.8 days, with no statistically significant difference between the 2 groups (*p* > 0.05). The postoperative hospitalization days in the T4 group ranged from 4 to 6 days, with a mean of 5 ± 1 days, while the postoperative hospitalization days in the mSSLF group ranged from 3.5 to 5.5 days, with a mean of 4.5 ± 1 days, indicating no statistically significant difference between the two groups (*p* > 0.05). The comparison of intraoperative conditions and postoperative recovery between the two groups is shown in Table 2.

**Table 2:** Perioperative outcomes by surgical group.^a^

Outcome	mSSLF group (*n* = 50)	T4-mesh group (*n* = 50)	*p-*value
Blood loss, ml	70 ± 10.2	90 ± 14.8	**0** **.** **03**
Operative time, min	50 ± 15.2	60 ± 14.8	**0** **.** **02**
Postoperative stay, d	4.5 ± 1	5 ± 1	0.65
Vascular Injury	0	1	0.319
Bladder or rectum Injury	0	0	–
Postoperative fever	3	2	0.24
Delayed hemorrhage	0	0	–
Infection	0	0	–
Abscessus	0	0	–
Catheterization	3.98 ± 0.8	4.4 ± 1.1	0.32

^a^ Values are given as mean ± SD or number (percentage), unless indicated otherwise. Bold value indicated *p* value was meaningful.

### Comparison of the improvements in POP-Q at the 2-year follow up in both groups

The anatomical repositioning of the POP-Q point was restored in both groups, and the postoperative TVL was prolonged in both groups compared with the preoperative period, but there were no statistically significant differences between the two groups during the 6-, 12-, and 24-month postoperative follow-ups. Among them, the difference in TVL and point C between the two groups was more significant but not statistically significant (*p* > 0.05), see in Table 3.

**Table 3:** Anatomic outcomes measured by POP-Q at three timepoints after surgery.^a^

POP-Q	mSSLF group (*n* = 50)	T4 group (*n* = 50)	*p-*value
6 mouths			
Aa	−2.5 ± 0.2	−2.4 ± 0.2	0.63
Ba	−2.7 ± 0.2	−2.6 ± 0.8	0.53
Ap	– 2.5 ± 0.4	−2.6 ± 0.3	0.36
Bp	−2.3 ± 0.3	−2.4 ± 0.2	0.28
C	−7.0 ± 0.5	−7.1 ± 0.4	0.56
TVL	7.1 ± 0.4	7.0 ± 0.5	0.61
12 mouths			
Aa	−1.5 ± 0.3	−1.4 ± 0.2	0.55
Ba	−1.4 ± 0.2	−1.5 ± 0.2	0.53
Ap	– 1.7 ± 0.5	−1.4 ± 0.6	0.57
Bp	−1.5 ± 0.4	−1.3 ± 0.4	0.36
C	−6.2 ± 0.5	−6.0 ± 0.6	0.43
TVL	6.1 ± 0.7	6.3 ± 0.6	0.52
24 mouths			
Aa	−1.0 ± 0.1	−1.3 ± 0.3	0.43
Ba	−0.9 ± 0.2	−1.1 ± 0.1	0.25
Ap	– 1.5 ± 0.3	−1.4 ± 0.4	0.56
Bp	−1.2 ± 0.3	−1.1 ± 0.3	0.64
C	−5.8 ± 0.7	−5.3 ± 0.4	0.45
TVL	5.9 ± 0.5	5.2 ± 0.7	0.55

^a^ Values are given as mean ± SD unless indicated otherwise. Bold value indicated *p* value was meaningful.

### Comparison of subjective symptom scores between the two groups

A remarkable improvement in functions and life quality was observed in the two groups after the procedures according to the PFDI-20 scores. POPDI-6 and UDI-6 scores also showed significant improvement compared to preoperative scores. However, there was no statistically significant difference between the two groups at the postoperative follow-up (*p* > 0.05). See Table 4.

**Table 4:** Functional outcomes measured by the PFDI-20 at the 2-year follow-up.^a^

Outcome	mSSLF group (*n* = 50)	T4 group (*n* = 50)	*p-*value
PFDI-20 score			
Preoperative	56.5 ± 24.8	53.1 ± 27.3	0.49
2-year follow-up	20.6 ± 10.5	19.7 ± 10.2	0.11
*p-*value	**<0.001**	**<0.001**	
POPDI-6 score			
Preoperative	20.8 ± 9.8	23.8 ± 10.8	0.76
2-year follow-up	9.2 ± 2.8	8.8 ± 1.3	0.06
*p-*value	**<0.001**	**<0.001**	
UDI-6 score			
Preoperative	12.4 ± 2.8	13.8 ± 3.8	0.76
2-year follow-up	8.8 ± 2.5	9.8 ± 1.3	0.06
*p-*value	**<0.001**	**<0.001**	

^a^ Values are given as mean ± SD unless indicated otherwise. Bold value indicated *p* value was meaningful.

### Comparison of postoperative complications in both groups

In this study, there were no patients with recurrence greater than stage 2 during the 1st year of follow-up, but during the 2nd year of follow-up, there were total 8 patients re-emergence of pelvic prolapse in 2 groups, 1 anterior pelvic recurrence and 2 posterior pelvic prolapse in the mSSLF group and 1 anterior pelvic prolapse, 2 mid-pelvic prolapse and 2 posterior pelvic prolapse in the T4 group. 4 patients in the T4 group presented with postoperative pain in the puncture area, which was significantly higher than that of the mSSLF group, while 3 patients in the T4 group presented with vaginal foreign body sensation at 6 months after surgery. In the mSSLF group, no cases of vaginal foreign body sensation were recorded. In the T4 group, 2 cases with exposed mesh were recorded at 6 months after surgery, both of which were located in the anterior wall near the dome, and the exposure range was 0.3–0.8 cm, and healed well after local trimming and estriol treatment. In contrast, no mesh erosion was reported in the mSSLF group. No difficulty in defecation was reported in either group after surgery. Painful intercourse: patients in the mSSLF group resumed sexual intercourse after surgery and did not experience painful intercourse. Among the 50 patients in the T4 group, 7 (14%) felt mild pain or discomfort during intercourse 6 months after surgery, the symptoms of 2 of these patients improved but the 5 other patients still had mild symptoms 12 months after surgery. The difference in the incidence of painful intercourse between the two groups was statistically significant (*p* < 0.05). In addition, 2 new cases of lower urinary tract symptoms in the T4 group after surgery, while no such cases were reported in the mSSLF group during the follow-up period. These results are presented in Table 5.

**Table 5:** Postoperative complications.^a^

	mSSLF group (*n* = 50)	T4 group (*n* = 50)	*p-*value
re-emergence prolapse ≥stage 2	3	5	0.29
Mid prolapse	0	2	**0** **.** **15**
Anterior prolapse	1	1	0.56
Posterior prolapse	2	2	0.14
Difficult defecation	0	0	–
New cases of lower urinary tract symptoms	0	2	0.15
postoperative pain	0	4	**0.02**
vaginal foreign body sensation	0	4	**0.04**
Exposure to mesh	0	2	0.15
Painful intercourse	0	7	**0.005**

^a^ Values are given as number (percentage) or number, unless indicated otherwise. Bold value indicated *p* value was meaningful.

## Discussion

At present, since patients require further improvement of the quality of life and the high recurrence rate after traditional surgical treatment, pelvic floor surgery accounts for 40% of general gynecological surgery (11, 12), which has attracted a high level of attention of relevant departments and experts. It is widely believed that surgical treatment of pelvic organ prolapse should not only focus on its therapeutic effect and reduce the recurrence rate after surgery, but more importantly the quality of life of the patients should be improved compared with the level before surgery. This is the ultimate goal and the most important challenge for the treatment of pelvic floor deficiency diseases.

The traditional classical SSLF procedures separate the pararectal space from the sacrospinous ligament by an incision made on the posterior vaginal wall and attaches the top of the prolapsed vagina to the sacrospinous ligament through sutures, restoring the relative position of the vagina on the pelvic floor, which not only allows the vagina to have a sufficient length and reconstructs the horizontal axis of the vagina, the procedure preserves the uterus and is suitable for patients who are sexually active (13). However, it is not suitable for patients with combined anterior pelvic prolapse and there is a risk of recurrence after surgery, with the most common being recurrence of anterior vaginal wall prolapse (14). Our modified procedure was able to preserve and improve on this classic surgical approach. First of all the access route used was modified to be able to reach the sacrospinous ligament through the paravaginal space, anterior to the peri-cervical ring, making the surgical route more optimal, and reducing hematoma, thus preventing anterior pelvic recurrence.

In addition, further studies on the anatomy of the pelvic floor has led to the enhanced understanding of the shortcomings of traditional surgery and there is now a unified consensus on the outcome of surgical treatment for POP (i.e., surgery has a support and reconstruction focus) (15). The modern surgical concept of pelvic floor reconstruction not only continues to adhere to the restoration of anatomical structures and related functions, but also requires the replacement of weak and damaged pelvic floor fascial tissues using reasonably applied alternative materials, in a manner that is accepted by all involved. The classic T4 prolift pelvic floor reconstruction procedure uses mesh placement, one pairs of anterior and one pair of posterior slings for fixation, simultaneous repair of multiple defective areas, reconstruction of pelvic floor structure and restoration of pelvic floor support function, with a high cure rate and low recurrence rate. However, since T4 pelvic floor reconstruction requires extracorporeal puncture to introduce the mesh band during the procedure, there are also challenges such as postoperative pain in the puncture area and tissue damage. In addition, the placement of the mesh poses the risk of mesh erosion.

Another more commonly performed procedure, Prosima pelvic floor reconstruction, does not require extracorporeal puncture and avoids the challenges caused by perforation, damage to the sacrospinous ligament and bladder, and postoperative pain in the puncture area. However, as the mesh is not fixed intraoperatively, it is only suitable for patients with symptomatic moderate pelvic organ prolapse, and its recurrence probability is higher than that of Prolifl pelvic floor reconstruction (16). Prosima requires vaginal balloon placement after surgery, which can lead to bleeding and necrosis of vaginal tissue or infection.

In this study, we reasonably applied a small amount of mesh to replace weak and damaged pelvic floor fascial tissue, fixed the posterior wall mesh to the sacrospinous ligament, and placed the anterior wall in the space between the anterior vaginal wall and the bladder. This modified procedure not only resulted in stronger mesh fixation than that of the Prosima pelvic floor reconstruction procedure, but also significantly reduced the separation gap and puncture space compared with T4 prolift pelvic floor reconstruction, which significantly reduces the incidence of intraoperative complications and the probability of recurrence, leading to a significant reduction in the incidence of intraoperative complications and the probability of recurrence. In our study, one case of more severe vascular injury occurred in the T4 mesh group, and closed-hole vascular injury was considered a possibility. It is well known that the pelvic floor structure and blood vessels are rich and complex, and it is difficult to treat injuries after they occur, and most of them are treated conservatively, which can easily induce discomfort such as hematoma, delayed infection, and nerve damage. Therefore, reducing the number of puncture points can reduce damage to surrounding tissue and blood vessel and greatly reduce the occurrence of surgical malign events.

Secondly, the incidence of sexual difficulties after pelvic floor reconstruction surgery has been reported to range from 2% to 15%, with a higher rate of sexual discomfort (17). Analysis of the resulting sexual difficulties have revealed that they may be related to postoperative vaginal contracture, hardening, and contracture of the patch too close to the anal levator complex. In addition, the placement of a large mesh affects the elasticity and tension of the vaginal mucosa, sex In our modified procedure, the size of the mesh used was reduced, and the upper arm of the mesh was placed without puncture, with less tension and significantly less postoperative sexual function than in the T4 group. In addition, the procedure was also performed using sacrospinous ligament suspension, which allowed for the relatively significant anatomical position during repositioning and very good apical vaginal support. Moreover, sacrospinous ligament suspension can better promote the stretching of the mesh during reconstruction, enhance support of the pelvic floor to a greater extent, reduce weaknesses, and improve the clinical cure rate, while reducing the occurrence of postoperative complications.

The incidence of patch erosion has been reported to be 4%–11% and is a complication unique to synthetic patches that mostly occurs within six months after surgery, and the occurrence of such complications is mostly related to the individual characteristics, the depth of mesh embedding, and rejection reaction (18–20). In the T4 group, the incidence was 4% but there was no mesh exposure reported in the modified procedure, which is thought to be due to the sacrospinous ligament suspension performed in this procedure to promote mesh extension during the reconstruction. In addition, a less amount of mesh was used for the modified surgery, which is beneficial for mesh exposure prevention.

Pelvic organ prolapse and stress urinary incontinence are both manifestations of female pelvic floor disorders that can be considered as different manifestations of the same disease at different times (21–23). The probability of new SUI after pelvic floor reconstruction is reported to be 8%–60%. These symptoms may be due to the urethra being obstructed preoperatively due to the severe prolapse of the organ, which has squeezed and distorted the urethra. Some patients also present with insidious stress incontinence, which gradually becomes apparent after pelvic floor reconstruction due to a change in the urethral angle (24, 25). In this study, two new cases of lower urinary tract symptoms of varying degrees were found in the T4 group during the postoperative follow-up period, while no new symptoms were found in the mSSLF group. Considering that following pure anterior pelvic floor reconstruction, only the anterior pelvic floor is reinforced, while pelvic pressure transmission may lead to secondary mid or posterior pelvic floor prolapse, while sacrospinous ligament suspension can effectively decrease weakness and reinforce the pelvic floor structure mesh group during the follow-up period, thus reducing the occurrence of incontinence. However, considering the association between the short follow-up period or the small number of cases included in the study population, further studies are needed to verify these results.

Modified sacrospinous ligament fixation using a transvaginal anterior wall route with promising short-term efficacy and safety, may be a feasible technique for the treatment of severe prolapse. Hence, additional studies with a larger number of patients and a longer follow-up period should be conducted.

## Data Availability

The original contributions presented in the study are included in the article/Supplementary Material, further inquiries can be directed to the corresponding author/s.
